# Detection of the third and fourth heart sounds using Hilbert-Huang transform

**DOI:** 10.1186/1475-925X-11-8

**Published:** 2012-02-14

**Authors:** Yi-Li Tseng, Pin-Yu Ko, Fu-Shan Jaw

**Affiliations:** 1 Institute of Biomedical Engineering, National Taiwan University, Taipei 10617, Taiwan; 2 Department of Electrical Engineering, National Taiwan University, Taipei 10617, Taiwan

**Keywords:** Third heart sound, Fourth heart sound, Hilbert-Huang Transform, time-frequency analysis, Phonocardiogram

## Abstract

**Background:**

The third and fourth heart sound (S3 and S4) are two abnormal heart sound components which are proved to be indicators of heart failure during diastolic period. The combination of using diastolic heart sounds with the standard ECG as a measurement of ventricular dysfunction may improve the noninvasive diagnosis and early detection of myocardial ischemia.

**Methods:**

In this paper, an adaptive method based on time-frequency analysis is proposed to detect the presence of S3 and S4. Heart sound signals during diastolic periods were analyzed with Hilbert-Huang Transform (HHT). A discrete plot of maximal instantaneous frequency and its amplitude was generated and clustered. S3 and S4 were recognized by the clustered points, and performance of the method was further enhanced by period definition and iteration tracking.

**Results:**

Using the proposed method, S3 and S4 could be detected adaptively in a same method. 90.3% of heart sound cycles with S3 were detected using our method, 9.6% were missed, and 9.6% were false positive. 94% of S4 were detected using our method, 5.5% were missed, and 16% were false positive.

**Conclusions:**

The proposed method is adaptive for detecting low-amplitude and low-frequency S3 and S4 simultaneously compared with previous detection methods, which would be practical in primary care.

## 1. Introduction

Auscultation has long been important for the diagnosis of heart diseases. Heart sounds heard by a stethoscope can be seen as mechanical instructions that indicate the operation of the cardiac system. The third and fourth heart sounds, which are two abnormal components of heart sounds during diastolic periods, have been found to have relationships with myocardial dysfunction [1-5]. The third and fourth heart sounds have been discovered over a century [6]. The third heart sound (S3) occurs in the rapid filling period of early diastole. It is often present in systolic dysfunction [7]. Abnormal S3 is considered to be caused by altered physical properties of ventricle or increased in the rate and volume of blood flow in the rapid filling phase during ventricle diastole [5]. Sometimes it occurs in children. However, the auscultation of S3 in adults, especially elders older than 40 years old, is abnormal and is connected with heart failure. The fourth heart sound (S4) occurs in late diastolic periods right before the first heart sound. The presence of S4 is due to the forceful contraction of the atria in an effort to overcome an abnormally stiff or hypertrophic ventricle [8]. It can be detected in patients with diseases of diminished left ventricular compliance, such as acute myocardial infarction or ischemia [9].

The importance of S3 and S4 has been notified early in 1970s [5]. In 1997, *M. Ishikawa et al*. discovered that the appearing of S4 during long-term follow-up of acute myocardial infarction may be a strong indicator of poor prognosis [1]. Later in 2006, *G. Marcus et al*. have indicated that S3 has high specificity and can be a marker of left ventricular dysfunction [3]. In 2009, *E. Lee et al*. has proved that patients without clinical ST criteria for ischemia developed new or increased-intensity S3 and S4 during percutaneous coronary intervention induced ischemia [2]. Therefore, the combination of using diastolic heart sounds with the standard ECG as a measurement of ventricular dysfunction may improve the noninvasive diagnosis of myocardial ischemia.

Several studies have verified the characteristics of S3 and have applied time-frequency methods to detect S3 [10,11]. However, there have been no efficient methods specific for S4 detection due to its lower amplitude and uncertain frequency. Time-frequency methods have already been used for heart sounds analysis [12], but mainly in the analysis of the first and second heart sounds [13,14]. These advanced signal processing methods, such as Short Time Fourier Transform, Wigner-Ville Distribution, and Wavelet transform, have some limitations [15,16]. The major disadvantage of the Short Time Fourier Transform is the resolution trade-off between time and frequency domain. The Wigner-Ville Distribution provides better resolution in both time and frequency domain, but its bilinear characteristic produces cross-term interferences. In recent years, the wavelet transform has become a widely used and versatile time-frequency method. The wavelet transform has variable time and frequency resolution, and it is able to carry out local analysis. These advantages made wavelet transform received considerable research attention. In 2005, *Hult et al*. have developed a wavelet-based method for recognition of S3 [17]. However, the wavelet transform is not an adaptive method. Once the mother wavelet function is generated, it cannot be modified again to adapt to non-stationary signals. As non-stationary signals with large varieties of amplitude and frequency, heart sounds with S3 and S4 are more suitable to be analyzed by the Hilbert-Huang transform (HHT). HHT has been used to analyze heart sound signals in previous studies [18,19], while most of these methods are proposed to classify normal heart sound components such as S1 and S2. The analysis of abnormal heart sound components are still in research. Hilbert-Huang transform is a time-frequency method proposed by *Norden Huang *in 1998 [20]. It is a powerful method in the analysis of non-stationary and nonlinear signals. The empirical mode decomposition of HHT can decompose heart sound signals adaptively to numbers of intrinsic mode functions, and Hilbert transform of these functions generates instantaneous frequency of signals. Hilbert-Huang transform provides fine resolution of three-dimensional time-frequency distribution of energy.

In this paper, we proposed a further extraction of the information of instantaneous frequency carried out by Hilbert-Huang transform. The extracted frequency-magnitude distributions have been clustered and further analyzed. Components of S3 and S4 in abnormal heart sound could be recognized and compared with normal heart sound. Performance of the method was estimated quantitatively.

## 2. Materials and Methods

**Figure 1:**
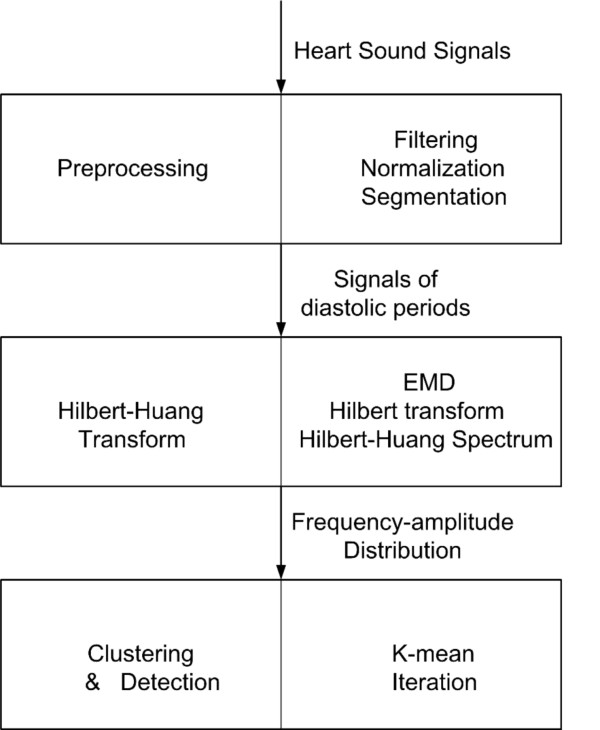
**Diagram of the recognition method**.

An adaptive-based algorithm was developed for the detection of S3 and S4 which are non-stationary signals with low amplitude and frequency. The schematic diagram of this recognition method was demonstrated in Figure 1. The proposed method could be divided into three steps: (1) Preprocessing, (2) Hilbert-Huang Transform and (3) Clustering and recognition. Details of these three steps were described in the following subsections.

### 2.1 Preprocessing

Heart sound signals recorded by electronic stethoscopes are often encompassed with high frequency noise, hence preprocessing is essential. As illustrated in Figure 1, the signals were filtered to eliminate the noise, and followed by normalization and segmentation. These steps were illustrated in the following:

*Filtering and Smoothing: *Since heart sound signals are mainly less than 600 Hz, a Butterworth low-pass filter designed by digital finite impulse response (FIR) was applied. Hilbert transform was then used to produce the envelope of the signals. The envelope was denoted as *x_envelope_*[*n*], where

(1) $$x_{envelope}\left[n\right]=\left|x\left[n\right]+j\times hilbert\left\{x\left[n\right]\right\}\right|,$$

and *x*[*n*] was the raw data of heart sound signals.

*Normalization: *The amplitude of different heart sound signals were all normalized and limited to the scale of [-1 1]. The equation of normalization is in the following:

(2) $$x_{norm}\left[n\right]=\frac{x_{envelope}\left[n\right]}{\text{max}\left|x_{envelope}\left[n\right]\right|}.$$

**Figure 2:**
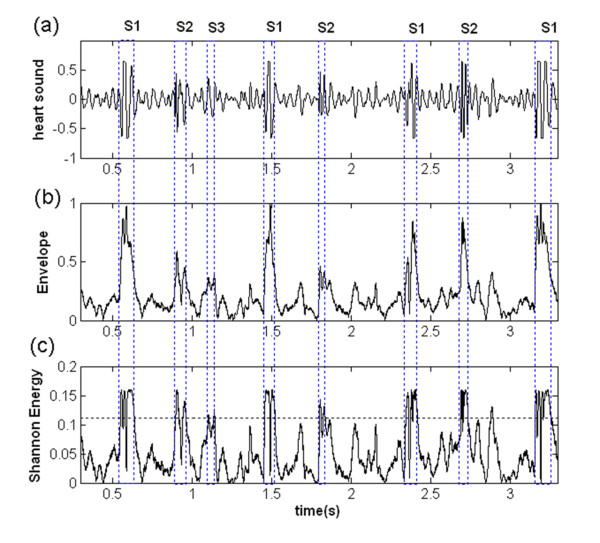
**The envelope (b) and Shannon Energy (c) of a heart sound record (a)**. The threshold was shown with a dashed line in (c).

An example of normalization and enveloping of an abnormal heart sound record (Figure 2(a)) was shown in Figure 2(b).

*Segmentation: *The heart sound signals should be segmented into cycles before processing. Four terms were recognized during this step: the first heart sound (S1), the second heart sound (S2), systolic period, and diastolic period. To detect these terms with noise interference rejection, Shannon energy (SE) of signals was used and calculated as follows [21-25]:

(3) $$SE\left[n\right]=-x_{norm}^{2}\left[n\right]\times \text{log}x_{norm}^{2}\left[n\right].$$

As shown in (3), the feature of Shannon energy was to suppress the low amplitude components of signal [22]. The main components, S1 and S2, would therefore be picked up. Compared with other algorithms, such as absolute value or Shannon entropy, Shannon energy would be better for segmenting of noisy heart sound signals [22].

The Shannon energy of the heart sound record with S3 was shown in Figure 2(c). The threshold value was initially set to 70% of maximal value. If the threshold is chosen upper than 70%, some S2 with lower peak would be excluded and missed. But for threshold lower than 70%, noise, murmur, or additional heart sound would easily be picked up and affect the results of S1 and S2 recognition. Nevertheless, higher threshold is rather better because it would be easier to re-pick the missing S2 than dealing with noises. S1 and S2 were recognized by three steps in the following [24]:

a. If two peaks higher than the threshold were detected within 50 ms, the one with lower energy was eliminated.

b. For every interval between peaks, an interval with shorter length than the previous interval was denoted as a systolic period, while the other one was a diastolic period. The uncertain intervals were annotated.

c. For those uncertain intervals, a secondary threshold was set to find S1 or S2 which probably not have been recognized.

These steps were applied to ensure that S1 and S2 could be picked out correctly. Systolic and diastolic periods were then recognized. S3 and S4 with larger amplitudes could also be detected during segmentation.

### 2.2 Hilbert-Huang transform

Instantaneous frequency and its magnitude of preprocessed heart sound signals were extracted by Hilbert-Huang transform (HHT). HHT was used to adaptively decompose the non-stationary and nonlinear signals and extract the instantaneous frequency. As illustrated in Figure 1, HHT consisted of two steps: Empirical mode decomposition (EMD) and Hilbert transform. EMD was used to adaptively decompose the signal into a series of intrinsic mode functions (IMFs). Hilbert transform was then carried out to acquire instantaneous frequency and amplitude and constitute the time-frequency-energy distribution, Hilbert-Huang spectrum, of the signal.

### 2.2.1 Empirical mode decomposition (EMD)

The heart sound signal was first decomposed to IMFs. To acquire the IMFs, local minima and maxima of the signal were found out. The envelopes of the local minima and maxima were formed by cubic spline fitting, respectively. Let *m_1_(t) *denoted as the average of these two envelopes, and the original signal was subtracted by *m_1_(t) *as follows:

(4) $$x\left(t\right)-m_{1}\left(t\right)=h_{1}\left(t\right).$$

Took *h_1_(t) *as a new signal and repeated the process described above until the resulting *h_1_(t) *met the criterion of the IMF [20]. The resulted signal was the first IMF defined as *c_1_(t)*, and the residual signal was *r*_1_*(t)*, where

(5) $$r_{1}\left(t\right)=x\left(t\right)-c_{1}\left(t\right).$$

The *r*_1_*(t) *was then considered as a new original signal and the iterative process was again executed to extract the IMFs until the *k*th residual signal *r_k_(t) *became a singular function, which meant that no more IMF could be further extracted. The *x(t) *was therefore expressed by

(6) $$x\left(t\right)=\overset{k}{\underset{i=1}{\sum }}c_{i}\left(t\right)+r_{k}\left(t\right),$$

and *c_1_(t) *to *c_k_(t) *were *k *IMFs of the signal.

EMD method would make the signal more symmetrical by eliminating the riding waves and decomposes the signal adaptively.

### 2.2.2 Hilbert transform

The second step of HHT, Hilbert transform, extracted the instantaneous frequency and amplitude of each IMF. Each component *c_i_(t) *of IMFs was Hilbert transformed, denoted by *y_i_(t)*, so:

(7) $$y_{i}\left(t\right)=\frac{1}{\pi }\int_{-\infty }^{\infty }\frac{c_{i}\left(\tau \right)}{t-\tau }d\tau .$$

The combination of *x_i_(t) *and *y_i_(t) *was an analytic signal z*_i_(t)*, where

(8) $$z_{i}\left(t\right)=x_{i}\left(t\right)+jy_{i}\left(t\right)=a_{i}\left(t\right)e^{j\theta_{i}\left(t\right)},$$

and *x_i_(t) *and *y_i_(t) *were respectively the real part and imaginary part of z*_i_(t)*. The amplitude and phase of z*_i_(t) *were defined by the following expressions:

(9) $$a_{i}\left(t\right)=\sqrt{x_{i}^{2}\left(t\right)+y_{i}^{2}\left(t\right)},$$

(10) $$\theta_{i}\left(t\right)={\text{tan}}^{-1}\left(\frac{y_{i}\left(t\right)}{x_{i}\left(t\right)}\right).$$

Since the definition of the instantaneous frequency was

(11) $$d\omega_{i}\left(t\right)=\frac{d\theta_{i}\left(t\right)}{dt},$$

the original signal could be expressed by

(12) $$x\left(t\right)=\overset{n}{\underset{i=1}{\sum }}a_{i}\left(t\right)e^{j\int \omega_{i}\left(t\right)dt}.$$

The instantaneous frequency and amplitude could therefore be acquired for further extraction, or simply formed a time-frequency plot, denoted as the Hilbert-Huang spectrum.

### 2.3 Clustering and Recognition

In this subsection, the relationship between instantaneous frequency and its amplitude were plotted and clustered. By correlating the clustered points with the original sound signal, the positions of S3 and S4 were labeled. For those possibly missing components, an iterative method was applied to enhance the accuracy.

**Figure 3:**
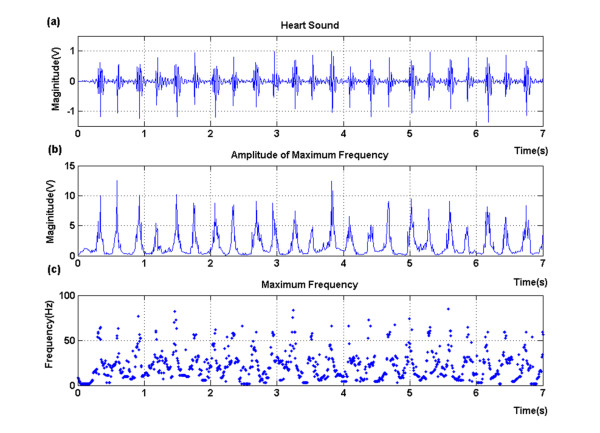
**A normal heart sound record (a)**. The magnitude (b) of the maximal instantaneous frequency (c) was indicated as well.

**Figure 4:**
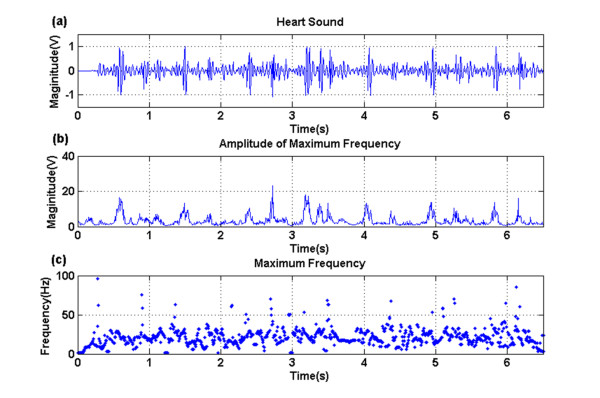
**An abnormal heart sound with the presence of the third and fourth heart sound (a)**. The magnitude (b) of the maximal instantaneous frequency (c) was indicated.

The components with the maximal amplitude of each instantaneous frequency were selected in (12). That is to say, as simplification we considered only the maximal contribution of frequency at a time. The extracted instantaneous frequency and its amplitude were shown in Figure 3 and 4. A normal heart sound signal was shown in Figure 3(a). There were two major components within a normal beat, the first heart sound (S1) and the second one (S2). In comparison to Figure 3(a), there were additional components, S3 and S4, during diastolic periods (S2-S1 interval) in Figure 4(a). The amplitudes of S3 and S4 were smaller than S1 and S2 with great varieties.

The maximal instantaneous frequency and its amplitude were therefore used to distinguish S3 and S4 from the baseline noise in the interval from S2 to S1. Figure 3(b) and 3(c) illustrated the magnitude of the maximal instantaneous frequency and the amplitude of the frequency, and so did Figure 4(b) and 4(c). Our data were sampled with a sampling frequency of 8000 Hz. Figure 3(c) indicated that the maximal instantaneous frequency of a normal heart sound was mainly lower than 100 Hz. The S1 and S2 were around 50 Hz. Components of the baseline signal were with low frequency, while there were a few numbers of points carried with larger frequency which might be caused by the baseline noise. However, Figure 4(c) indicated that there were components with larger instantaneous amplitude or frequency during diastolic periods in an abnormal record with S3 and S4.

**Figure 5:**
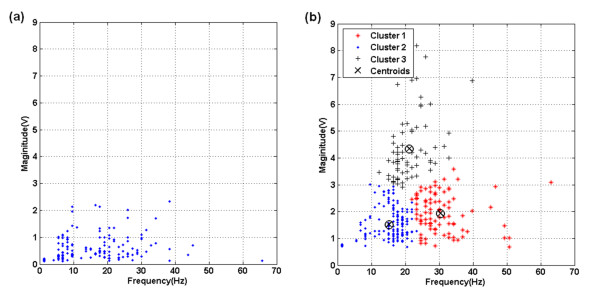
**Frequency-Magnitude plot of the diastolic interval - (a) Normal heart sound**. (b)Abnormal heart sound with S3 and S4 after cluster analysis.

Since there were great variations both in the amplitude and in the instantaneous frequency of S3 and S4, these two parameters should to be considered simultaneously when we were trying to verify whether there are S3 or S4 in a record. Therefore, we plotted the maximal instantaneous frequency and its magnitude of the S2-S1 interval of the above two heart sounds in Figure 5. The number of points depends on the resolution of Hilbert spectrum. Figure 5(a) showed the relationship between the maximal instantaneous frequency and its magnitude in a normal heart sound, whereas Figure 5(b) indicated an abnormal one with S3 and S4 during the S2-S1 interval. It is obvious that the frequency-magnitude distribution of Figure 5(b) was more diverse.

K-mean algorithm was then used as cluster analysis of frequency-magnitude distribution. Also shown in Figure 5(b) with different symbol, the distributed data could be divided into three groups: Normal points with low amplitude and frequency, uncertain points, and abnormal points with high frequency or amplitudes. The group with the fewest number of points was denoted as the last one. These points in the abnormal group could then be projected to the original Hilbert spectrum and the time when abnormal points occurred would be verified. If the abnormal signals occur periodically right before S1, they could be denoted as S4. In contrast, S3 occurs periodically after S2.

An iterative recognition method was then applied for detecting those components that are possibly missing. Since S3 and S4 are various both in amplitude and frequency, an adaptive detection method might occasionally misjudge noise signals as heart sound components or vice versa. The iterative recognition method examined whether the detected points were periodically occurring. For those positions where S3 or S4 should have been detected, the method determined if there is a missing point. Consequently, S3 and S4 of heart sound signals could be recognized separately with enhanced accuracy.

## 3. Results

Heart sound records from Cardiac Auscultatory Recording Database (CARD) of Johns Hopkins University [26] were used to verify the recognition accuracy of the proposed method. The CARD database contains up to 800 records, and about 15 records include S3 or S4. Thirteen recording samples were included and the patients' information was provided in Table 1. There were also other databases with one or two samples with S3 or S4. However, for the consideration of their distinct sources of recording and the validation of signals, we only used samples from CARD database as the concern of reliability. These samples with S3 or S4 were recorded in the position where extra heart sounds were best heard.

**Table 1:** Information of patients including their ID, age, gender, recording position, the presence of S3 and S4

Patient ID	age	gender	position	S3	S4
**56**	18	M	LLSB^1^	no	yes
**139**	13	M	LLSB	yes	no
**189**	11	F	APEX LMSB^2^	occasional	yes
**202**	22	F	LLSB	yes	no
**405**	15	M	APEX	yes	no
**485**	6	F	APEX	yes	no
**498**	6	F	APEX	yes	no
**509**	9	M	APEX	no	yes
**516**	17	M	LMSB	yes	no
**830**	15	M	LMSB	yes	no
**907**	20	M	APEX	yes	no
**1169**	14	M	APEX	yes	no
**1204**	22	M	LLSB	yes	no

^1 ^LLSB = Left Lower Sternal Border

^2 ^LMSB = Left Middle Sternal Border

**Figure 6:**
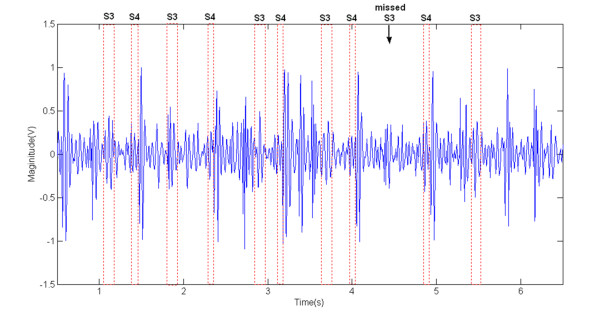
**Abnormal heart sound signal with S3 and S4 labeled**.

An example from one of the patients with S3 and S4 after utilizing the iterative recognition method was shown in Figure 6. For these recording samples, the total cycles for recognition and the results of detection were demonstrated in Table 2. Using the proposed method with iterative recognition, 90.3% of heart beat cycles with S3 were identified, 9.6% were missed and 9.6% were false positive. For S4, 94.4% were detected, 5.5% were missed and 16% were false positive (FP).

**Table 2:** Number of cycles where S3 and S4 are recognized, missed or false recognized

Patient ID	S3			S4			Total Cycles
		
	recognized	missed	False	recognized	missed	false	
**139**	5	0	0				5
**202**	2	1	0				3
**405**	6	0	1				6
**485**	6	0	0				6
**498**	5	1	0				6
**516**	5	2	1				7
**830**	5	0	0				5
**907**	5	0	2				5
**1169**	7	0	0				7
**1204**	6	0	2				6
**189**	4	2	0	5	1	0	6
**56**				7	0	1	7
**509**				5	0	0	5

The overall performance of the algorithm was evaluated by sensitivity and precision. The detected and missed component were denoted as true positive (TP) and false negative (FN). The sensitivity and precision of the method was therefore calculated as follows:

(13) $$sensitivity=\frac{TP}{TP+FN}$$

(14) $$precision=\frac{TP}{TP+FP}.$$

The sensitivity of the detection method was 90.4% and 94.5% for S3 and S4, and the precision of S3 and S4 were 90.4% and 85.5%, respectively.

## 4. Discussion

The aim of this research was to automatically recognize S3 and S4 in an abnormal heart sound. This method further extracted information such as the maximal instantaneous frequency and amplitude from a time-frequency spectrum of Hilbert-Huang transform. The signals were adaptively decomposed and transformed. The extraction could provide information of a heart sound signal by retaining only the main frequency component. Then the cluster analysis compared the signal in the same record. Using the proposed method, the sensitivity for S3 and S4 were 90.4% and 94.5%, and the precision were 90.4% and 85.5%, respectively. The sensitivity of S4 detection was better than S3. With the adaptive method based on time-frequency analysis, the algorithm eliminated the influence of noise and body movement. The effect of great variance of S3 and S4 could also be eliminated. Automated recognition of S3 and S4 within the same method would therefore be feasible.

However, the existence of extra sounds during diastolic period, such as diastolic murmur or noise produced by the electronic stethoscope, would still contribute to misjudgments. Since the method includes the whole period of diastole, the interference of these extra sounds could not be eliminated. Nevertheless, using the whole period of diastole is essential since the occurring time of S3 and S4 is different from patient to patient. The interference of noise could be minimized by the proposed relative clustering analysis method when the noise and murmur are no larger than S3 and S4.

The low amplitude sometimes makes S3 and S4 indistinguishable from background noise, which would be one of the limitations of using them as diagnostic parameters. This characteristic increase the difficulties for clinicians to hear and judge the presence of S3 and S4, thus reduce the reliability of diagnosing by these two components. Some recent studies have also evaluated the relationship between the level of physician experience and accurate auscultation of heart sound [3]. These investigations mostly claimed that clearly heard S3 and S4 could be seen as markers with high specificity associated with left ventricular dysfunction and improve the detection rate for the patients who are nondiagnostic by standard ECG [1,2].

Although some studies still demonstrated that the pathological influence of S3 and S4 was under estimation [27,28], heart sounds has already been encompassed in acoustic cardiography which combines ECG and sound information to diagnose myocardial ischemia [2,4,13]. The method we proposed is especially aimed for early detection and auto-alarm for some heart diseases, such as left ventricle dysfunction, congenital heart failure, or myocardial ischemia. For these kinds of applications, non-invasive fast and flexible algorithms would be facile to be implemented in an ambulance or remote home health care system. Several time-frequency methods have also been developed for diastolic heart sound analysis in recent years [24]. In comparison with previous detection methods, our method is adaptive for non-stationary heart sound signal. Therefore it could detect low-amplitude and low-frequency S3 and S4 simultaneously and would be practical in primary care.

## 5. Conclusion

The proposed method based on Hilbert-Huang transform is adaptive for detecting low-amplitude and low-frequency S3 and S4, which are seen as the early symptoms of myocardial dysfunction. Further extraction of the instantaneous frequency was carried out by Hilbert-Huang transform. The extracted frequency-magnitude distributions was clustered and analyzed. Components of S3 and S4 in abnormal heart sound could therefore be recognized. Performance of the method was estimated using the CARD database. In comparison with previous detection methods, S3 and S4 could be detected simultaneously using the proposed method and the performance was quantitatively evaluated.

## Competing interests

The authors declare that they have no competing interests.

## Authors' contributions

YL carried out the algorithm design, implementation, and wrote the paper; PY worked on the signal processing of heart sound segmentation; FSJ contributed to discussion and suggestions of the topic and manuscript writing. All authors read and approved the final manuscript.
